# Promise of Smartphone-Enabled Teleconsultation for Global Cancer Prevention

**DOI:** 10.1200/GO.20.00424

**Published:** 2020-09-17

**Authors:** Paul C. Pearlman

**Affiliations:** ^1^ Center for Global Health, National Cancer Institute, Rockville, MD

Few low- or middle-income countries (LMICs) have comprehensive cancer prevention strategies in place. Moreover, implementing such strategies requires substantial investments in health systems, both in terms of facilities and human capital, as well as the will and ability of people to pay for preventive services.^1^ As LMICs increasingly bear a major share of the burden of cancer (as of 2018, nearly 60% of cancer incidence and > 70% of cancer mortality), creative and cost-effective solutions for early detection and downstaging are direly needed to reduce the burden on fragile health systems and increase access to care.^2^ Individuals living far from tertiary care centers or belonging to marginalized populations are at increased risk for late diagnoses and poor outcomes. To this end, two publications in this journal have recently leveraged smartphone-based mobile platforms that rely on a teleconsultation approach to improve cancer screening at the community level in Tanzania.^3,4^

A plethora of factors, including new and emerging technologies, study methods, data sources, and analytic techniques, are expanding the toolkit of possible solutions to bolster cancer prevention and control in low-resource settings globally.^5^ Nonetheless, as a percentage of spending on cancer research across all sectors, current investment in the development of new technologies and the validation of emerging technologies that may be promising for early detection and screening is likely insufficient.^6^ Moreover, the introduction of new technologies has a worrying tendency to exacerbate health disparities. To achieve the potential of new technologies, it is essential to strive toward full access among the target population, implement robust quality control to reduce the harms associated with screening, and deliver the right intervention at each step in the cascade of events that encompass screening.^6^

For good reason, telehealth and mHealth, the use of mobile technologies to provide health support to patients or technical support to service providers, have received a lot of attention from the global health community.^7-9^ This is especially true as the world copes with the COVID-19 pandemic, and systems rapidly roll out a legion of new remote health services.^10^ According to the International Telecommunications Union, > 95% of the global population is covered by mobile networks, meaning mHealth-based interventions require little investment in new infrastructure, making them a direct, reliable, and cost-effective approach to increasing access to care in remote settings and among underrepresented populations in health systems.^11^ Moreover, as broadband Internet coverage increases and new technologies such as 5G networks reduce latency, a wide variety of digital health solutions will increasingly be available to the majority of the world.^5^ For all of these reasons, support for research and development of resource-appropriate technologies, including mHealth, continues to be a focal area for the National Cancer Institute Center for Global Health.^12-14^

Increasingly sophisticated cameras built into even the lower-end smartphones have enabled the acquisition of high-quality digital images of gross anatomy that can conceivably be used as part of a cancer screening program. To this end, Rubagumya et al^3^ introduce a teleconsultation approach to reduce cancer health disparities and improve cancer outcomes for people with albinism in Tanzania, supported by an app titled NgoziYangu (ie, “my skin” in Swahili). This report is particularly exciting because the work addresses the often-late presentation of skin cancers in a stigmatized group experiencing sociocultural and economic disparities. Moreover, because the risk of developing skin squamous cell carcinoma is estimated to be 1,000-fold higher in those with albinism than in the general African population, this work naturally stratifies the intervention to the highest-risk group, an important consideration in a region with insufficient dermatologists.

The investigators rely on a store-and-forward approach to analyze smartphone-acquired images of potentially malignant lesions, avoiding existing computer-assisted classification approaches because of their reported low negative predictive values compared with expert human review. The authors also rely on consensus across a panel of expert reviewers to determine guidance for follow-up and/or referral for diagnostic workup. Premalignant and benign lesions were not biopsy confirmed in this study, so the accuracy of the test could not be confirmed. Although there is reason in the literature to suspect reasonable performance, negative predictive value is a key performance indicator, and the lack of it is a significant limitation of this study. Additionally, it would be interesting to characterize intraobserver variability among the expert reviewers to better understand the knowledge and training requirements for successful implementation as well as to provide context for scaling up the intervention, potentially through a cascade training model (ie, a training process whereby the trainee becomes the subsequent trainer), as described by Yeates et al.^4^ As this work develops, it will be exciting to gauge the impact of this tool on reducing diagnostic delay.

Yeates et al^4^ have developed an approach for improving the quality of visual inspection with acetic acid (VIA)–based cervical cancer screening, titled Smartphone Enhanced VIA (SEVIA). The principal contribution of this work is the use of the smartphone platform to maintain quality control through a teleconsultation approach for training, monitoring, and evaluating frontline health workers performing VIA that doubles as a tool to train future consultants through a cascade training approach. The need for robust quality control is paramount, because VIA is likely to be the dominant screening approach for cervical cancer for some time and is further needed to assess the cervix for ablative therapy when treating dysplasia.

The cascade training approach is a massive contribution toward taking this intervention to scale. The near real-time feedback that the health provider conducting the examination receives through SEVIA improves the knowledge and skill of that frontline worker. By quantitatively evaluating concordance with the expert reviewers, the program coordinators are provided an objective method for assessing when this individual no longer requires expert review and are potentially qualified to become reviewers themselves. Moreover, as the authors suggest, random auditing of SEVIA results can allow for an entry point for refresher training, further bolstering quality control efforts.

Yeates et al^4^ assess the performance of SEVIA by looking at the change in the mean VIA positivity rate over a 6-month window at each of their 24 sites compared with the year before implementation. Because VIA positivity rates in Tanzania have consistently been lower than expected based on global estimates, the increase in VIA positivity (compared with Tanzania’s reference standard) is a surrogate for an increase in screening quality. Similar to the Rubagumya et al^3^ study, histopathologic confirmation would be desirable to assess accuracy. Nonetheless, the SEVIA approach almost certainly results in substantial quality improvements.

To achieve full access among the target population, scalability is crucial. The teleconsultation approaches used in both of these studies can create the environment and data sets necessary for training program staff, as occurred in the Yeates et al^4^ study. Smartphones are a potentially affordable enabling technology for these interventions, because they more than adequately replace digital single-lens reflex cameras/colposcopes for imaging the cervix and dedicated optical imaging equipment, such as dermoscopes, for skin lesions. In addition, although in 2017 the average smartphone in Tanzania cost roughly 16% of an average person’s income, this price point may be acceptable for targeted health programs, even if costs do not come down with increasing access.^15^ Neither study included a detailed cost analysis; however, Yeates et al^4^ estimate the cost of SEVIA at < US$1 per test. The NgoziYangu intervention can be assumed to be of equally low cost. In addition, the expert review enabled by these smartphone platforms is essential in ensuring the quality of the test and reducing the harms associated with screening. Finally, challenges associated with real-time evaluation and loss to follow-up remain, although increasing mobile broadband coverage may partially mitigate the former, and future expansions on these mobile apps themselves may be incorporated into patient education and/or navigation plans. In essence, both of these approaches have a high likelihood of fully realizing the potential of their screening technologies.

The technologies developed by Rubagumya et al^3^ and Yeates et al^4^ both have the potential to further support the development of related technologies. As an example, both could be leveraged to create and strengthen digital image repositories for ancillary studies. Such repositories could eventually be rich data sources for machine learning and artificial intelligence, and the smartphone platforms themselves could be used for refinement and fielding of resulting algorithms. The applications of such tools for clinical decision support could streamline the teleconsultation process and help mitigate some of the challenges described here.

Ultimately, the confidence researchers and providers have in digital technologies must be mirrored in the community. Ethical, legal, and social implications research concerning data security, confidentiality, and acceptability of such interventions are all of great importance. Continued investment in implementation science research for such technologies as well as sustained community engagement will determine long-term success. Technology alone will not leapfrog over the need for skilled and knowledgeable personnel, and is no panacea for health systems, but there are reasons to be optimistic.
